# The case for investment in eye health: systematic review and economic modelling analysis

**DOI:** 10.2471/BLT.23.289863

**Published:** 2023-10-30

**Authors:** Brad Wong, Kuldeep Singh, Bryce Everett, Kieran S O’Brien, Thulasiraj Ravilla, Rohit C Khanna, Heidi Chase, Kevin D Frick

**Affiliations:** a Mettalytics, 23 Philip St, South Golden Beach 2483, New South Wales, Australia.; b Seva Foundation, Delhi, India.; c Department of Economics, College of Arts and Sciences, University of San Francisco, San Francisco, United States of America (USA).; d Department of Ophthalmology, Institute for Global Health Sciences, University of California San Francisco, San Francisco, USA.; e Lions Aravind Institute of Community Ophthalmology, Aravind Eye Care System, Madurai, India.; f Gullapalli Pratibha Rao International Centre for Advancement of Rural Eye care, L V Prasad Eye Institute, Hyderabad, India.; g Seva Foundation, Berkeley, USA.; h Johns Hopkins Carey Business School, Johns Hopkins University, Baltimore, USA.

## Abstract

**Objective:**

To assess how the returns on investment from correcting refractive errors and cataracts in low- and middle-income countries compare with the returns from other global development interventions.

**Methods:**

We adopted two complementary approaches to estimate benefit-cost ratios from eye health investment. First, we systematically searched PubMed® and Web of Science™ on 14 August 2023 for studies conducted in low-and-middle-income countries, which have measured welfare impacts associated with correcting refractive errors and cataracts. Using benefit-cost analysis, we compared these impacts to costs. Second, we employed an economic modelling analysis to estimate benefit-cost ratios from eye health investments in India. We compared the returns from eye health to returns in other domains across global health and development.

**Findings:**

We identified 21 studies from 10 countries. Thirteen outcomes highlighted impacts from refractive error correction for school students. From the systematic review, we used 17 out of 33 outcomes for benefit-cost analyses, with the median benefit-cost ratio being 36. The economic modelling approach for India generated benefit-cost ratios ranging from 28 for vision centres to 42 for school eye screening, with an aggregate ratio of 31. Comparing our findings to the typical investment in global development shows that eye health investment returns six times more benefits (median benefit-cost ratio: 36 vs 6).

**Conclusion:**

Eye health investments provide economic benefits with varying degrees based on the intervention type and location. Our findings underline the importance of incorporating eye health initiatives into broader development strategies for substantial societal returns.

## Résumé

**Objectif:**

Comparer les retours sur investissement d'une correction des défauts de réfraction et des cataractes dans les pays à revenu faible et intermédiaire avec ceux d'autres interventions liées au développement mondial.

**Méthodes:**

Nous avons adopté deux approches complémentaires afin d'estimer les rapports coûts/avantages d'un investissement dans la santé oculaire. Tout d'abord, nous avons passé au crible PubMed® et Web of Science™ le 14 août 2023, à la recherche d'études menées dans des pays à revenu faible et intermédiaire sur l'impact d'une correction des défauts de réfraction et des cataractes, puis nous avons procédé à une analyse coûts/avantages dans le but de comparer cet impact aux dépenses engendrées. Ensuite, nous avons effectué une analyse par modélisation économique afin de déterminer les rapports coûts/avantages des investissements dans la santé oculaire en Inde. Enfin, nous avons comparé les retours sur investissement dans ce domaine avec ceux d'autres domaines liés à la santé et au développement à l'échelle mondiale.

**Résultats:**

Nous avons identifié 21 études portant sur 10 pays. Parmi les résultats obtenus, 13 mettaient en évidence l'impact qu’une correction des défauts de réfraction représentait pour les étudiants. À l'issue de notre revue systématique, nous avons utilisé 17 des 33 résultats pour nos analyses coûts/avantages, le rapport médian s'élevant à 36. L'approche par modélisation économique pour l'Inde a débouché sur des rapports coûts/avantages allant de 28 pour les centres de la vision à 42 pour le dépistage des troubles de la vue en milieu scolaire, avec une proportion globale de 31. En comparant nos résultats avec les dépenses classiques en matière de développement mondial, nous avons constaté qu'investir dans la santé oculaire permettait de multiplier les avantages par six (rapport coûts/avantages médian: 36 contre 6).

**Conclusion:**

Les investissements dans la santé oculaire s'accompagnent d'avantages économiques à différents niveaux, en fonction du lieu et du type d'intervention. Nos résultats montrent à quel point l'intégration d'initiatives de santé oculaire au sein de stratégies de développement plus vastes est importante dans la réalisation de bénéfices considérables pour la société.

## Resumen

**Objetivo:**

Evaluar la rentabilidad de la inversión en la corrección de los defectos de refracción y las cataratas en los países de ingresos bajos y medios en comparación con la rentabilidad de otras intervenciones de desarrollo a escala mundial.

**Métodos:**

Se adoptaron dos enfoques complementarios para estimar la relación coste-beneficio de la inversión en salud ocular. En primer lugar, se realizó una búsqueda sistemática en PubMed® y Web of Science™ el 14 de agosto de 2023 sobre estudios realizados en países de ingresos bajos y medios, que han medido los efectos en el bienestar asociados a la corrección de los errores de refracción y las cataratas. Mediante el análisis beneficio-coste, se compararon estos efectos con los costes. En segundo lugar, se empleó un análisis de modelos económicos para estimar la relación beneficio-coste de las inversiones en salud ocular en la India. Asimismo, se compararon las rentabilidades de la salud ocular con las de otros sectores de la salud y el desarrollo mundiales.

**Resultados:**

Se identificaron 21 estudios de 10 países. Trece resultados destacaron los efectos de la corrección de los defectos de refracción en estudiantes. A partir de la revisión sistemática, se utilizaron 17 de los 33 resultados para los análisis de beneficio-coste, en los que la mediana de esta relación fue de 36. El enfoque de modelación económica para la India generó relaciones beneficio-coste que van de 28 para los centros oftalmológicos a 42 para el cribado ocular en las escuelas, con una relación agregada de 31. Si se comparan los resultados obtenidos con la inversión típica en desarrollo mundial, se observa que la inversión en salud ocular genera una rentabilidad seis veces mayor (relación beneficio-coste mediana: 36 frente a 6).

**Conclusión:**

Las inversiones en salud ocular aportan beneficios económicos de diversa magnitud según el tipo de intervención y la ubicación. Los resultados obtenidos destacan la importancia de incorporar las iniciativas de salud ocular a estrategias de desarrollo más amplias para obtener una rentabilidad social significativa.

## ملخص

**الغرض:**

تقييم كيفية عقد مقارنة بين عوائد الاستثمار من تصحيح الأخطاء الانكسارية، وحالات الكتاراكت (إعتام عدسة العين) في الدول ذات الدخل المنخفض والدخل المتوسطة، والعوائد من تدخلات التنمية العالمية الأخرى.

**الطريقة:**

انتهجنا أسلوبين متكاملين لتقدير نسب التكلفة إلى الفائدة من الاستثمار في صحة العيون. قمنا أولاً بالبحث بشكل منهجي في كل من PubMed®‎ وWeb of Science™‎ في 14 أغسطس/آب 2023، عن الدراسات التي تم إجراؤها في الدول ذات الدخل المنخفضة والدخل المتوسط، والتي قامت بقياس تأثيرات الرفاهية المرتبطة بتصحيح الأخطاء الانكسارية وحالات الكتاراكت (إعتام عدسة العين). وباستخدام تحليل الفائدة إلى التكلفة، قمنا بمقارنة هذه التأثيرات بالتكاليف. وقمنا ثانياً بالاستعانة بتحليل وضع النماذج الاقتصادية ، لتقدير نسب الفائدة إلى التكلفة من استثمارات صحة العيون في الهند. وقمنا بمقارنة العوائد من صحة العيون، بالعوائد في المجالات الأخرى للصحة والتنمية العالمية.

**النتائج:**

قمنا بتحديد 21 دراسة من 10 دول. أوضحت ثلاث عشرة نتيجة التأثيرات الناجمة عن تصحيح الأخطاء الانكسارية لدى طلاب المدارس. ومن المراجعة المنهجية، قمنا باستخدام 17 نتيجة من أصل 33 نتيجة لتحليلات الفائدة إلى التكلفة، حيث بلغ متوسط نسبة الفائدة إلى التكلفة 36. وأدى أسلوب وضع نموذج اقتصادي في الهند إلى تكوين نسب للفائدة إلى التكلفة تتراوح بين من 28 لمراكز الرؤية، إلى 42 لفحص العيون في المدارس. مع بنسبة إجمالية قدرها 31. إن مقارنة النتائج لدينا مع الاستثمار النموذجي في التنمية العالمية، تشير إلى أن الاستثمار في صحة العيون يعود بفوائد أكثر بستة أضعاف (متوسط نسبة الفائدة إلى التكلفة: 36 مقابل 6).

**الاستنتاج:**

توفر الاستثمارات في صحة العيون فوائد اقتصادية بدرجات متفاوتة بناءً على نوع التدخل والموقع. تؤكد النتائج لدينا على أهمية دمج مبادرات صحة العيون في استراتيجيات التنمية الأوسع، وذلك لتحقيق عوائد مجتمعية كبيرة.

## 摘要

**目的:**

评估在低收入和中等收入国家进行矫正屈光不正和治疗白内障投资的回报与实施其他全球发展干预措施的回报相比的情况。

**方法:**

我们采用了两种互补的方法来估算眼部健康投资的成本效益比。首先，我们于 2023 年 8 月 14 日系统地检索了 PubMed® 和 Web of Science™，以查找在低收入和中等收入国家进行的相关研究，这些研究衡量了与矫正屈光不正和治疗白内障相关的福利影响。使用效益成本分析法，我们将这些影响与成本进行了比较。其次，我们采用经济模型分析来估算在印度进行眼部健康投资的效益成本比。我们将眼部健康的投资回报与全球健康和发展中其他领域的回报进行了比较。

**结果:**

我们确定了来自 10 个国家的 21 项研究。其中 13 项研究结果强调了屈光不正矫正对在校学生的影响。在系统评价中，我们采用了 33 个研究结果中的 17 个进行效益成本分析，效益成本比的中位数为 36。印度的经济建模分析法产生的效益成本比从 28（视力中心）到 42（学校眼部筛查）不等，总比值为 31。将我们的研究结果与全球发展的典型投资进行比较，显示进行眼部健康投资的回报效益是后者的六倍（效益成本比的中位数：36 对比 6）。

**结论:**

根据实施干预类型和地点的不同，进行眼部健康投资可产生不同程度的经济效益。将保护眼部健康倡议纳入更广泛的发展战略可获得可观的社会回报，我们的研究结果强调了这一重要性。

## Резюме

**Цель:**

Оценить, насколько окупаемость инвестиций в коррекцию аномалий рефракции и катаракты в странах с низким и средним уровнем дохода сопоставима с окупаемостью других мероприятий в области глобального развития.

**Методы:**

Для оценки соотношения затрат/выгоды от инвестиций в здоровье глаз использовались два взаимодополняющих подхода. Во-первых, по состоянию на 14 августа 2023 г. в базах данных PubMed® и Web of Science™ был выполнен систематический поиск исследований, проведенных в странах с низким и средним уровнем дохода, в которых оценивалось влияние на качество жизни, связанное с коррекцией аномалий рефракции и катаракты. С помощью анализа соотношения выгоды/затрат было проведено сравнение влияния и затрат. Во-вторых, для оценки соотношения выгоды/затрат от инвестиций в здоровье глаз в Индии был использован анализ экономического моделирования. Полученные результаты в области здоровья глаз сравнивались с результатами в других областях глобального здравоохранения и развития.

**Результаты:**

Было выявлено 21 исследование в 10 странах. Тринадцать исходов свидетельствуют о влиянии коррекции аномалий рефракции на школьников. По результатам систематического обзора для анализа соотношения выгоды/затрат использовались 17 из 33 исходов, при этом медиана соотношения выгоды/затрат составила 36. В результате экономического моделирования для Индии были получены соотношения выгоды/затрат в диапазоне от 28 для офтальмологических центров до 42 для проверки зрения в школах, при этом суммарное соотношение составило 31. При сравнении полученных результатов с типовыми инвестициями в глобальное развитие видно, что инвестиции в здоровье глаз приносят в шесть раз больше выгоды (среднее соотношение выгоды/затрат: 36 по сравнению с 6).

**Вывод:**

Инвестиции в здоровье глаз обеспечивают экономические выгоды различной степени в зависимости от типа и места проведения вмешательства. Полученные результаты подчеркивают важность включения мероприятий по охране здоровья глаз в более широкие стратегии развития для получения значительной отдачи для общества.

## Introduction

Although vision impairment affects 2.2 billion people,^1^ eye health services are underfunded and undervalued in global health policy. Notably, *Transforming our world: the 2030 agenda for sustainable development* lacks any reference to eye health.^2^ The World Health Organization (WHO) estimates a funding gap of 14.3 billion United States dollars (US$) to address preventable vision loss, and notes poor integration of eye services within existing health systems.^1^ Estimates from 2014–2018 shows that eye health investment from bilateral, multilateral and private philanthropic institutions was US$ 102 million annually, less than 0.06% of development spending.^3^ This limited acknowledgement of eye health as an important area for investment may have contributed to the lack of progress against the World Health Assembly goal of reducing avoidable vision impairment by 25% between 2010 and 2019.^4^

One potential reason is that eye health investment, especially in addressing uncorrected refractive error, falls short of being one of the best health investments when comparing incremental cost per disability-adjusted-life-year (DALY) averted.^3^^,^^5^^,^^6^ Reducing DALYs at lowest cost is a widely accepted criterion for prioritizing health investments. However, this approach overlooks the potential additional benefits of interventions, such as productivity gains and broader welfare improvements. Eye health interventions, which may have low DALYs averted yet prevent significant productivity losses, are consequently at a disadvantage when using a cost-per-DALY-averted criterion. For instance, the estimated disability weight for blindness indicates an 18.7% annual health loss.^7^ However, productivity loss, even just considering employment, averages 30.2% for those with blindness and moderate or severe visual impairment.^3^^,^^8^ Considering refractive error, near and mild vision impairment disability weights indicate a 1.1% or 0.3% annual health loss, respectively.^7^ Studies providing glasses in agriculture,^9^ manufacturing,^10^ and school settings^11^^–^^14^ suggest annual productivity losses that are approximately five to 20 times greater than corresponding health losses. Therefore the value of eye health interventions might be better reflected in productivity and other welfare gains, rather than by averted DALYs alone.^15^

The purpose of this paper is to demonstrate how productivity and other welfare outcomes can be incorporated into economic evaluations of eye health investment, focusing on refractive error and cataracts, the two largest sources of visual impairment.^1^ By conducting a systematic review, we identified studies from low-and middle-income countries which have measured productivity, income, learning and other welfare impacts associated with improving vision from refractive error and cataracts. Using the outcomes of the identified studies, we calculated the benefit-cost ratio to assess the welfare benefits of the intervention against its costs. One significant advantage of benefit-cost analysis is that it encompasses various benefits, not limited to health impacts.^16^ Additionally, we employed an economic modelling analysis to estimate benefit-cost ratios from eye health investments in India.

## Methods

### Systematic review

The protocol for the systematic review was registered and published on Open Science Framework.^17^ We conducted a literature search in PubMed® and Web of Science™ on 14 August 2023 with no language restrictions. Box 1 shows the inclusion criteria. We excluded studies that focused solely on health-related outcomes like DALYs, quality-adjusted-life-years, mental health outcomes and health expenditure.

Box 1 **Inclusion criteria for studies used to calculate benefit-cost ratios for interventions improving vision from refractive error and cataracts**

The study compared two groups cross-sectionally with and without refractive error or cataracts, where an impact variable is defined as the dependent variable in a regression, and there is an attempt to control for endogeneity such as instrumental variable design or propensity score matching; or a study that provided eyeglasses or cataract surgery to the study population, estimating outcomes over time with or without comparison to groups which were not provided with eyeglasses or cataract surgery.

The study measured and reported quantitative impacts in the following non-health welfare outcomes: (i) employment, productivity, income, consumption, expenditure, gross margins, profit and revenue at the individual, household or enterprise level; or (ii) academic performance in school children.

The study was conducted in a low-or middle-income country

The study was published between 1 January 2001 and 31 December 2022 inclusive.

Two authors independently screened titles and abstracts to identify articles for full-text assessment. They then assessed the selected full-text articles to confirm that studies met the inclusion criteria. They resolved disagreements through discussion. If impossible, a third author acted as arbitrator. We also consulted recent reviews at the intersection of eye health and economics or education,^3^^,^^15^^,^^18^^–^^20^ and reference lists of studies meeting inclusion criteria in our systematic review.

We extracted data about the study population, the intervention and study country, as well as information necessary to conduct the benefit-cost analyses. All outcomes used in the benefit-cost analyses were assessed using the Cochrane risk-of-bias assessment relevant to the study design. Publication bias was assessed using a funnel plot. More details are available in the online repository.^21^

### Estimating benefit-cost ratios

We adopted two complementary approaches to estimating benefit-cost ratios from eye health investment. The first approach used results of studies identified via the systematic review, where researchers had documented differences in the productivity, income or learning impacts between groups with and without visual impairment, including those with corrected vision. Using individual studies provides greater confidence in the magnitude of impacts and benefit-cost ratios, particularly when estimated using experimental approaches. 

For the second approach, we used an economic modelling analysis of benefits, along with detailed micro-costing data to estimate the benefit-cost ratios from implementation of four screening interventions at scale in India. The economic modelling approach facilitates the exploration of scaled interventions; however, the benefit-cost ratios reported might be less precise.

For both approaches, we conducted benefit-cost analysis from a societal perspective and adopted a common set of assumptions to facilitate comparability. Costs and benefits were discounted at a 3% rate, following published guidelines.^16^ We assumed that eyeglasses would last for three years for adult populations and one year for school children. We assumed that the benefits of cataract surgery would last for the rest of the beneficiary’s life. We sourced mean life expectancy of the study populations from World Health Organization (WHO) life tables.^22^

Where we adopted costs or benefits from the same country in a different year, we converted values to the same currency year using gross domestic product (GDP) deflators from the World Bank Development Indicators.^23^ Generally, values were reported in US$. Before making inflation adjustments, we converted prices to local currency using the exchange rate at the time the costs or benefits were assessed. Where we adopted costs and benefits from different countries, we used a cost-transfer approach which assessed the magnitude of cost in purchasing-power-parity (PPP) terms from the initial country as a percentage of GDP PPP, and applied that ratio for the same costs in the target country.

We compared the benefit-cost ratio distribution to a recent two-year project aimed to identify best-buy interventions in global health and development,^24^ and the Copenhagen Consensus Center library of 652 benefit-cost analyses of interventions.^25^ We also compared our estimated ratios to representative benefit-cost ratios for noncommunicable disease^26^ and nutrition interventions (online repository).^21^ We chose noncommunicable disease interventions because many eye health conditions are categorized as noncommunicable diseases. For nutrition, we chose these interventions, because their benefits might not be fully represented by traditional health-related cost-effectiveness metrics. Like eye health, the positive impacts of nutrition interventions can manifest in areas like current productivity,^27^ future productivity^28^ and learning.^29^

#### Individual studies

In each analysis, we assessed productivity, income and learning benefits, measures that are easily converted into monetary units for benefit-cost analysis. We also sourced costs from the programme, if reported. Where costs were unavailable, we identified costs by searching PubMed® and Web of Science™ for articles describing the same intervention conducted in the same country, or if that proved infeasible, we identified costs from other countries including a recent review on the costs of treatment.^15^ Where we used costs from another country, we prioritized countries from the same region before adopting costs from alternative regions. Box 2 provides an example of estimating the benefit-cost ratio of providing eyeglasses to tea-pickers in India.

Box 2 **Example of estimating benefit-cost ratios of an eye intervention from individual studies**

In Assam, India, providing eyeglasses to correct presbyopia in tea-pickers led to substantial productivity improvements: 5.25 kg more tea picked per day than a control group without corrected presbyopia.^9^ Over the 11-week harvesting season, with five working days per week, the amount of extra tea picked is therefore 289 kg per worker. Tea prices in Assam were no less than US$ 2.3 per kg in the period of the study (June to October 2017).^30^ The provision of eyeglasses yielded a productivity increase with an estimated market value of US$ 651 per year, or US$ 1842 for three years at a 3% discount rate. The cost of providing the eyeglasses was US$ 10.2 per person, including delivery. To this we add case-finding costs of US$ 8.0 and patient costs of US$ 1.2,^31^ resulting in a total cost of US$ 19.2. Comparing costs to benefits results in a benefit-cost ratio of 96.

US$: United States dollars.

In practice, our benefits were usually narrow, incorporating only one benefit that was reported in the study, while costs were more comprehensive, implying conservative benefit-cost ratios. We calculated ranges using reported confidence intervals (CIs) on benefit parameters. Further details on estimations from each study are available in the online repository.^21^

As per the pre-registered protocol,^17^ we refrained from including multiple benefit-cost ratios for the same study populations. We prioritized impacts measured using the most rigorous methods, those with longest follow-up time, or those closest to the intervention's general equilibrium effect. We conducted a sub-group analysis examining benefit-cost ratios from studies that did not present a serious risk of bias. 

#### Economic model

We based the economic model on two recently published studies from India: an economic modelling study of welfare losses associated with blindness and moderate and severe visual impairment;^32^ and a micro-costing analysis of interventions delivered at scale by six eye health providers.^31^ We chose this example because the costing data allowed for analysis of eye health interventions delivered at scale. The micro-costing analyses were performed on four case-finding strategies used by six Indian eye health providers, screening 2.3 million people in a year.^31^ Values were reported as annualized figures in 2020 US$, assuming a 3% discount rate.

We estimated benefits, costs and benefit-cost ratios using data and parameters from the studies. The model included more than 30 parameters, the most important are noted in Table 1, and the full list is available in the online repository.^21^ By simultaneously varying parameters across 10 000 Monte Carlo simulations, we calculated probabilistic estimates using both STATA (StataCorp LLC, College Station, United States of America) and Microsoft Excel (Microsoft Corp., Redmond, USA). These steps reflect probabilistic sensitivity analyses conducted in the original studies upon which we derived the benefit-cost analysis.^31^^,^^32^ The simulation allows us to report 95% CIs on benefit-cost ratios.

**Table 1:** Key parameters of benefit-cost model of four case-finding strategies, India

Parameter	Point estimate	Distribution and parameters for probabilistic sensitivity analysis
Discount rate	3%^16^	Uniform, 0%–8%^32^
Avoided loss in employment (moderate or severe visual impairment or blind)	30.2% reduction in employment for those aged 15–64^3^^,^^8^	Uniform, 19.5%–43.5%^32^
Avoided mortality (moderate or severe visual impairment)	1.26: 10-year all-cause mortality risk ratio relative to no visual impairment^3^^,^^33^	Gaussian, mean: 1.26; SD: 0.06^3^^,^^33^
Avoided mortality (blind)	1.90: 10-year all-cause mortality risk ratio relative to no visual impairment^3^^,^^33^	Gaussian mean: 1.90; SD: 0.26^3^^,^^33^
Improved productivity in employment	Avoided 20% productivity loss^9^^,^^34^^–^^36^	Uniform: 17%–23%^9^
Avoided caregiver costs (moderate or severe visual impairment)	5% of productive time for one person^37^	Uniform: 2.5%–10.0%
Avoided caregiver costs (blind)	10% of productive time for one person^37^	Assumed as twice the draw for moderate or severe visual impairment
Productivity improvement in domestic work (moderate or severe visual impairment or blind)	20% loss of productivity in household, non-market activities with value of loss estimated at 50% of wages^38^	Productivity loss equal to draw from productivity loss in employment; value of time: uniform: 25%–75%^38^
Increase in test scores from the provision of eyeglasses	0.18 standard deviation improvement^11^^–^^13^	Uniform: 0.11–0.23^11^^–^^13^
Compliance rate of eyeglasses use, adults	0.6^39^	Uniform: 0.4–0.8
Compliance rate of eyeglasses use, children	0.3^40^	Uniform: 0.2–0.4
Patient costs per screening, US$	1.2^41^	Gaussian mean: 1.2; SD: 0.18^41^
Patient costs per cataract surgery, US$	19.9^42^	Gaussian mean: 19.9; SD: 4.77^42^
Provider costs, case finding (US$ per treatment initiated)	8.0–29.3, varies by intervention and condition^31^	Follows probabilistic sensitivity analysis in Wong et al.^31^
Provider costs, one pair of eyeglasses, US$	4.8^31^	Uniform: 3.3–5.6^31^
Provider costs per cataract surgery, US$	71.0^31^	Uniform: 42.6–106.5^31^

SD: standard deviation; US$: United States dollars.

Notes: Summary of key parameters used in the benefit-cost model. Any reported currencies are in 2020 figures.

We estimated benefits for interventions targeting the general population (vision centres, eye camps and door-to-door screening) using a welfare model.^32^ The welfare includes benefits of increased employment, lower mortality risk, productivity gains in employment, welfare gains for the non-employed and avoided caregiver costs. The benefits for school screening are construed more narrowly as learning gains, where a standard deviation increase in test scores is associated with a 20% increase in adult income.^43^ Costs are estimated as the sum of patient and provider costs. Patient costs include the cost to access screening, indirect costs of receiving cataract surgery, and periodic eyeglasses repurchase costs as necessary. Provider costs include the costs of case finding and treatment. Further detail on the estimation is available in the online repository.^21^

## Results

### Systematic review

**Fig. 1:**
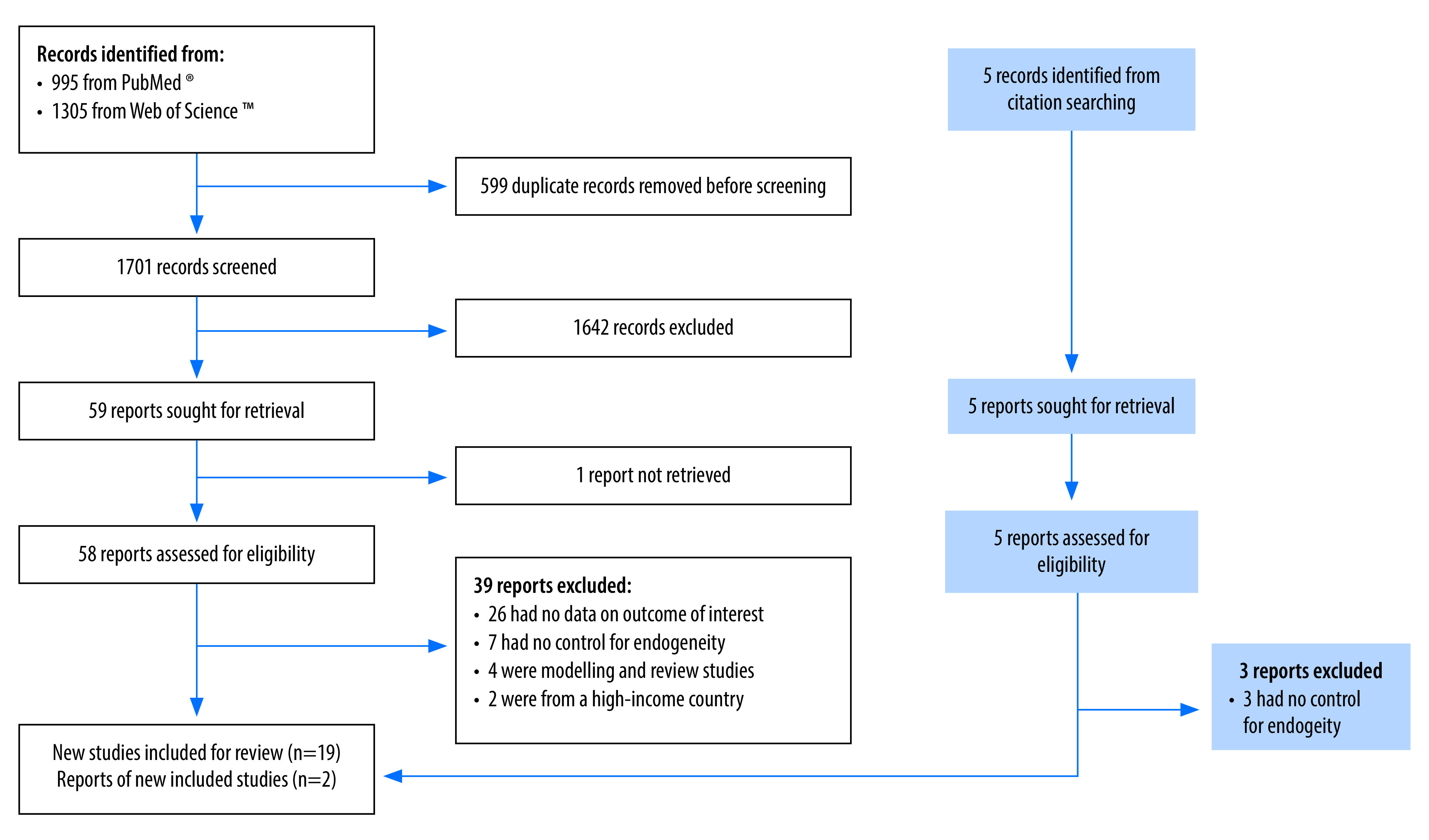
Selection of studies to be included in the analysis of eye health investment in low- and middle-income countries

The search yielded 1701 unique studies of which 59 full-text studies were consulted after screening titles and abstracts. Of these studies, 19 studies^9^^–^^13^^,^^34^^,^^44^^–^^56^ met the inclusion criteria (Fig. 1). Two additional studies^14^^,^^57^ meeting the inclusion criteria were identified via other methods, resulting in a final sample of 21 studies. These studies include 33 relevant productivity and welfare outcomes from 10 countries (Bangladesh, Cambodia, China, Ethiopia, India, Kenya, Pakistan, Philippines, South Africa and Viet Nam). Table 2 presents information on the studies and study outcomes.

**Table 2:** Empirical literature reporting productivity and welfare impacts of visual impairment identified in the systematic review

Study	Study design, site; year of implementation	Intervention description	Study population and sample size	Outcome for use in benefit-cost analysis	Risk of bias assessment^a^
Kuper et al., 2008^53^	Population-based case-control study Nakuru district, Kenya; 2005–2006	Cases: Cataract surgery (implied) Control: No intervention	Cases: Individuals with cataract aged ≥ 50 years with pinhole corrected visual acuity < 6/24 in the better eye Control: Age and gender matched individuals without visual impairment Cases: *n* = 142 Control: *n* = 75	Cases had lower monthly per capita expenditure (US$ 24) compared to controls (US $30)	Serious risk
Kuper et al., 2008^53^	Population-based case-control study Satkhira district, Bangladesh; 2005–2006	Cases: Cataract surgery (implied) Control: No intervention	Cases: Individuals with cataract aged ≥ 50 years with pinhole corrected visual acuity < 6/24 in the better eye Control: Age and gender matched individuals without visual impairment Cases: *n* = 216 Control: *n* = 279	Cases had lower monthly per capita expenditure (US$ 18) compared to controls (US$ 25)	Serious risk
Kuper et al., 2008^53^	Population-based case-control study Negros Island and Panay Islay, Philippines; 2005–2006	Cases: Cataract surgery (implied) Control: No intervention	Cases: Individuals with cataract aged ≥ 50 years with pinhole corrected visual acuity < 6/24 in the better eye Control: Age and gender matched individuals without visual impairment in the same cluster Cases: *n* = 238 Control: *n* = 180	Cases had lower monthly per capita expenditure (US$ 23) compared to controls (US$ 30)	Serious risk
Polack et al., 2008^46^	Population-based case-control study Nakuru district, Kenya; 2005–2006	Cases: Cataract surgery (implied) Control: No intervention	Cases: Individuals with cataract aged ≥ 50 years with pinhole corrected visual acuity < 6/24 in the better eye (*n* = 139) Control: Age and gender matched individuals without visual impairment (*n* = 124)	Cases: Maximum 14% (3.4/24 hours) of time spent on productive activities;^b^ 27% (38/139 people) require assistance in activities Controls: 24% (5.8/24 hours) of time spent on productive activities;^b^ 3% (4/124 people) require assistance in activities	Serious risk
Polack et al., 2008^46^	Population-based case-control study Satkhira district, Bangladesh; 2005–2006	Cases: Cataract surgery (implied) Control: No intervention	Cases: Individuals with cataract aged ≥ 50 years with pinhole corrected visual acuity < 6/24 in the better eye (*n* = 217) Control: Age and gender matched individuals without visual impairment (*n* = 280)	Cases: Maximum 13% (3.1/24 hours) of time spent on productive activities;^b^ 47% (102/217 people) require assistance in activities. Controls: 21% (5/24 hours) of time spent on productive activities;^b^ 9% (25/278 people) require assistance in activities	Serious risk
Polack et al., 2008^46^	Population-based case-control study Negros Island and Panay Islay, Philippines; 2005–2006	Cases: Cataract surgery (implied) Control: No intervention	Cases: Individuals with cataract aged ≥ 50 years with pinhole corrected visual acuity < 6/24 in the better eye (*n* = 238) Control: Age and gender matched individual(s) without visual impairment (*n* = 163)	Cases: Maximum 19% (4.6/24 hours) of time spent on productive activities;^b^ 22% (52/238 people) require assistance in activities. Controls: 25% (6/24 hours) of time spent on productive activities;^b^ 9% (15/163 people) require assistance in activities	Serious risk
Kuper et al., 2010^44^	Prospective cohort study with matched controls Nakuru district, Kenya; 2006	Exposed: Provision of free cataract surgery and transport to hospital Unexposed: No intervention	Exposed: Individuals with cataract aged ≥ 50 years with pinhole visual acuity < 6/24 in the better eye (*n* = 65) Unexposed: Age and gender matched individuals without visual impairment (*n* = 56)	After one year, per capita monthly household expenditure had increased from US$ 22 to US$ 30 in the exposed group and had increased from US$ 35 to US 36 in the unexposed group	Serious risk
Kuper et al., 2010^44^	Prospective cohort study with matched controls Satkhira district, Bangladesh; 2005–2006	Exposed: Provision of free cataract surgery and transport to hospital Unexposed: No intervention	Exposed: Individuals with cataract aged ≥ 50 years with pinhole corrected visual acuity < 6/24 in the better eye (*n* = 99) Unexposed: Age and gender matched individual(s) without visual impairment (*n* = 222)	After one year, per capita monthly household expenditure had increased from US$ 16 to US$ 23 in exposed group and had decreased from US$ 24 to US$ 23 in unexposed group	Serious risk
Kuper et al., 2010^44^	Prospective cohort study with matched controls Negros Island and Panay Islay, Philippines; 2005–2006	Exposed: Provision of subsidized cataract surgery and reimbursed transport costs Unexposed: No intervention	Exposed: Individuals with cataract aged ≥ 50 years with pinhole corrected visual acuity < 6/24 in the better eye (*n* = 99) Unexposed: Age and gender matched individuals without visual impairment (*n* = 152)	After one year, per capita monthly household expenditure had increased from US$ 24 to US$ 45 in the exposed group and had increased from US$ 32 to US$ 36 in the unexposed group	Serious risk
Polack et al., 2010^45^	Prospective cohort study with matched controls Nakuru district, Kenya; 2006	Exposed: Provision of free cataract surgery and transport Unexposed: No intervention	Exposed: Individuals with cataract aged ≥ 50 years with pinhole visual acuity < 6/24 in the better eye (*n* = 139) Unexposed: Age and gender matched individual(s) without visual impairment (*n* = 124)	After one year: 1 hour and 5 minutes more time spent on productive activities per day; Decrease in percentage of cases requiring assistance with activities from 25% to 12%	Serious risk
Polack et al., 2010^45^	Prospective cohort study with matched controls Satkhira district, Bangladesh; 2005–2006	Exposed: Provision of free cataract surgery and transport to hospital Unexposed: No intervention	Exposed: Individuals with cataract aged ≥ 50 years with pinhole corrected visual acuity < 6/24 in the better eye (*n* = 217) Unexposed: Age and gender matched individual(s) without visual impairment (*n* = 280)	After one year: 1 hour and 20 minutes more time spent on productive activities per day; Decrease in percentage of cases requiring assistance with activities from 43% to 19%	Serious risk
Polack et al., 2010^45^	Prospective cohort study with matched controls Negros Island and Panay Islay, Philippines; 2005–2006	Exposed: Provision of subsidized cataract surgery and reimbursed transport costs Unexposed: No intervention	Exposed: Individuals with cataract aged ≥ 50 years with pinhole corrected visual acuity < 6/24 in the better eye (*n* = 238) Unexposed: Age and gender matched individual(s) without visual impairment (*n* = 163)	After one year: 1 hour and 51 minutes more time spent on productive activities per day; Decrease in percentage of cases requiring assistance with activities from 23% to 1%	Serious risk
Finger et al., 2012^34^	Prospective cohort study, no control group Tamil Nadu, India; 2009–2010	Free cataract surgery and transport	Individuals with cataract (visual acuity < 6/60), aged ≥ 40 years, classified as poor and without prior cataract surgery; *n* = 294	After one year, average monthly household income had increased by 531 Indian rupees	Serious risk
Hannum & Zhang, 2012^48^	Cross-sectional survey of eyeglasses wearers with propensity score matched controls Gansu, China; 2004	Provision of eyeglasses to those with visual impairment (implied)	Junior high school students aged 13–16 years; Wearing spectacles: *n* = 94 (literacy test) or *n* = 106 (math and language test) Non-wearers: *n* = 1398 (literacy test) or *n* = 1450 (math and language test)	Treatment effect on the treated: Those wearing glasses had higher academic achievement, 0.34 SD improvement in literacy test scores; 0.26 SD improvement in math test scores and 0.13 SD improvement in language test scores	Moderate risk
Danquah et al., 2014^49^	Prospective cohort study with matched controls Satkhira district, Bangladesh; 2005–2006	Exposed: Provision of free cataract surgery and transport to hospital Unexposed: No intervention	Exposed: Individuals with cataract aged ≥ 50 years with pinhole corrected visual acuity < 6/24 in the better eye; (*n* = 56) Unexposed: Age and gender matched individuals without visual impairment; (*n* = 142)	After 6 years, per capita monthly household expenditure had increased from US$ 14.95 to US$ 23.29 in exposed group and had decreased from US$ 26.90 to US$ 24.29 in unexposed group	Serious risk
Danquah et al., 2014^49^	Prospective cohort study with matched controls Negros Island and Panay Islay, Philippines; 2005–2006	Exposed: Provision of subsidized cataract surgery and reimbursed transport costs to hospital Unexposed: No intervention	Exposed: Individuals with cataract aged ≥ 50 years with pinhole corrected visual acuity < 6/24 in the better eye; (*n* = 51) Unexposed: Age and gender matched individuals without visual impairment; (*n* = 91)	After 6 years, per capita monthly household expenditure had increased from US$ 21.73 to US$ 38.90 in exposed group and had increased from US$ 29.01 to US$ 37.40 in unexposed group	Serious risk
Essue et al., 2014^50^	Prospective cohort study, no control group; Hue, Binh Dinh, Vinh Long, and Thai Binh provinces, Viet Nam; 2011	Free cataract surgery	Individuals with cataract aged ≥ 18 years with a visual acuity ≥ 6/18 in the better eye; *n* = 381	After one year, the annual household income had increased by US$ 271 (51% increase)	Serious risk
Joseph, 2014^57^	Prospective cohort study with control group Thiruvananthapuram district, Kerala, India; No year reported	Exposed: Provision of free corrective eyeglasses for students with myopia Unexposed: Students without myopia	Rural and urban primary school students; Exposed *n* = 185 Unexposed: equated group; no other information reported	After five months, total test scores had increased from 20.5 to 27.1 in exposed group and from 26.4 to 27.5 in unexposed group	Serious risk
Ma et al., 2014^12^	Cluster RCT Tianshui prefecture, Gansu province and Yulin prefecture, Shaanxi province, China; 2012	Treatment: Provision of free eyeglasses following eye examination Control: Prescription for eyeglasses sent to parents with free eyeglasses provided at endline	Students at rural primary schools in grades 4–5 with uncorrected visual acuity of ≤ 6/12 in either eye Treatment: *n* = 1153 Control: *n* = 1036	After one school year, the intervention group had higher academic achievement, 0.11 SD improvement in test scores relative to the control group	Low risk
Ma et al., 2014^12^	Cluster RCT Tianshui prefecture, Gansu province and Yulin prefecture, Shaanxi province, China; 2012	Treatment: provision of vouchers for eyeglasses Control: Prescription for eyeglasses sent to parents with free eyeglasses provided at endline	Students at rural primary schools in grades 4–5 with uncorrected visual acuity of ≤ 6/12 in either eye Treatment: *n* = 988 Control: *n* = 1036	After one school year, the intervention group had non-significant increase in academic achievement, 0.04 SD improvement in test scores relative to the control group	Low risk
Glewwe et al., 2016^11^	Cluster RCT Two counties of Gansu province, China; 2004	Treatment: Eye examinations with provision of free eyeglasses Control: Students were screened for vision problems, but did not receive eyeglasses^c^	Students with poor vision in grades 4–6 in rural primary schools: Treatment: *n* = 1528 Control: *n* = 1001 (Regression results include 16 373 students with good vision in both treatment and control schools)	After one school year, the intervention group had higher difference in academic achievement, 0.11 SD improvement in test scores relative to the control group	Some concerns
Naidoo et al., 2016^10^	Prospective cohort study, no control group KwaZulu-Natal, South Africa; No year reported	Provision of free eye eyeglasses to address presbyopia	Textile workers aged ≥ 40 years performing near vision tasks in clothing factories (*n* = 268)	After 6 months, a 6.4% increase in production passing quality assurance per day	Serious risk
Ma et al., 2018^13^	Cluster RCT Yongshou county, Gansu province, China; 2014	Teacher screening followed by referral to vision centre to address myopia in students; The treatment group received early referral and the control group received late referral	Students at rural primary schools in grades 4–6 with uncorrected visual acuity of ≤ 6/12 (Snellen) in either eye Treatment: *n* = 433 Control: *n* = 516	4–6 months after referral to vision centre, the intervention group had higher academic achievement, 0.25 SD improvement in test scores relative to the control group	Some concerns
Reddy et al., 2018^9^	Individual RCT Assam, India; 2017	Treatment: Eye examination with provision of eyeglasses for presbyopia Control: Eye examination with deferred provision of eyeglasses for presbyopia	Tea pickers aged ≥ 40 years with near visual acuity ≤ 6/12 at 40 cm (presbyopia in both eyes) Treatment: *n* = 376 Control: *n* = 375	During the 11-week harvest season, treatment group had a 5.25kg higher average daily weight of tea picked, a 21.7% relative increase, compared to control group	Low risk
Glick et al., 2019^51^	Prospective cohort study with control group Amhara region, Ethiopia; 2012	Exposed: Free cataract surgery Unexposed: No intervention	Exposed: Individuals who were bilaterally blind from cataract, defined as presenting visual acuity in the better eye < 20/400 (*n* = 426) Unexposed: Blind individuals who were ineligible for cataract surgery (*n* = 471)	12-month follow up: difference-in-difference: 3.1% higher per capita consumption	Moderate risk
Nie et al., 2020^55^	Cluster RCT Shaanxi province, China; 2014	Treatment: Eye examination and provision of free eyeglasses Control: Eye examination with prescription provided. Eyeglasses provided after end of study	Grade 7–8 junior high school students Treatment: *n* = 476 Control: *n* = 434	After one school year, treatment group had 0.141 higher SD in test scores and 2.1 percentage point fewer drop outs compared to the control group	Some concerns
Ma et al., 2021^14^	Cluster RCT Gansu and Shaanxi province, China; 2013	Treatment: Eye examination and provision of free eyeglasses Control: Participants received a prescription for eyeglasses	Grade 5 students attending public primary schools Treatment: *n* = 1702 Control: *n* = 1763	After one school year, the intervention group had higher academic achievement, 0.14 SD improvement in math test scores relative to the control group	Some concerns
Ma et al., 2021^14^	Cluster RCT Jiangsu province, China; 2014	Treatment: Eye examination and provision of free eyeglasses Control: Participants received a prescription for eyeglasses	Grade 5 students attending migrant primary schools Treatment: *n* = 2163 Control: *n* = 2246	After one school year, 0.046 SD increase in math test scores in the treatment group relative to the control group	Some concerns
Du et al., 2022^47^	Cluster RCT; Rural county areas of Tianshui city, Gansu province, China; 2012	Treatment: Eye examinations with free eyeglasses delivered to schools four weeks later Control: Eye examinations with prescription for free eyeglasses	Students at rural primary schools in grades 4–5 with uncorrected visual acuity ≤ 6/12 in either eye; Treatment: *n* = 383 Control: *n* = 379	After one school year, the intervention group had higher academic achievement, 0.123 SD improvement in test scores relative to the control group	Some concerns
Du et al., 2022^47^	Cluster RCT; Rural county areas of Yulin city, Shaanxi province, China; 2012	Treatment: Eye examinations with free eyeglasses delivered to schools four weeks later Control: Eye examinations with prescription for free eyeglasses	Students at rural primary schools in grades 4–5 with uncorrected visual acuity ≤ 6/12 in either eye; Treatment: *n* = 716 Control: *n* = 609	After one school year, the intervention group had higher academic achievement, 0.032 SD improvement in test scores relative to control group	Some concerns
Latif et al., 2022^54^	Prospective cohort study, no control group Lahore, Pakistan; No year reported	Eye examinations and provision of free corrective eyeglasses	High school students (grade 6–10) across five local unit of an administrative division *n* = 253)	Higher academic achievement average test scores increased from 56.39 to 60.27 in public schools (equivalent to 0.29 SD improvement)	Serious risk
Sagemüller et al., 2022^56^	Cross-sectional survey with propensity score matching rural workers in the Cambodian agricultural sector, Cambodia; 2017–2018	Exposed: Provision of eyeglasses for visual impairment (implied) Unexposed: No intervention	Exposed: Workers with visual impairment (*n* = 179) Unexposed: Wokers without visual impairment (*n* = 76)	Treatment effect on the treated: Individuals with poor vision have US$ 630 lower farm profit compared to matched sample with good vision	Moderate risk
Huang & Chen, 2023^52^	Cross-sectional fuzzy regression discontinuity China Education Panel Survey, China; 2014–2015	Provision of eyeglasses to those with visual impairment (implied)	Grade 8 students (*n* = 3987)	Treatment effect on the treated: Those who wore glasses had higher academic achievement, 0.24 SD improvement in math test scores, 0.27 SD improvement in Chinese test scores	Low risk

RCT: randomized controlled trial; SD: standard deviation; US$: United States dollars.

^a^ RCTs were assessed using the Cochrane risk of bias tool for RCTs, which has three categories: low risk of bias, some concerns, high risk of bias. Observational studies were assessed using the Cochrane risk of bias tool for observational studies which has four categories: low risk of bias, moderate risk of bias, serious risk of bias and critical risk of bias.

^b^ Productive activities are household work, paid work, or work for own use.

^c^ Several townships were not compliant and provided control students with eyeglasses. These townships were excluded from the statistical analysis in the study’s preferred results.

Thirteen outcomes report on impacts associated with refractive error correction for school students. The remaining outcomes address household welfare impacts of cataract surgery, or productivity impacts of workers after refractive error correction. Seven studies (10 outcomes) were randomized controlled trials (RCTs); 14 studies (23 outcomes) were non-randomized observational studies. In terms of geographic coverage, 28 study outcomes were from Asian populations and five from sub-Saharan Africa. 

Of the outcomes, we categorized four as having a low risk of bias, 10 as having a moderate risk of bias or showing some concerns, and the remaining 19 as having a serious risk of bias. We did not identify evidence of publication bias for a sub-group of studies focusing on school screening (online repository).^21^

### Benefit-cost ratios

Of the 33 outcomes identified from the systematic review, 17 were used in the benefit-cost analyses. Fourteen outcomes described impacts of participants in the Cataract Impact Study conducted in Bangladesh, Kenya and the Philippines in 2004–2005.^44^^–^^46^^,^^58^ We only used three outcomes in the benefit-cost analysis to avoid over representation of the same study populations. Several papers reported results of RCTs offering school screening and free eyeglasses for primary grade students in the Chinese provinces Gansu and Shaanxi in 2012 and 2013.^12^^,^^14^^,^^47^ The information provided in these papers was unclear on whether these three study populations represented separate or overlapping groups. We therefore report only the benefit-cost ratio associated with the median impact of these three studies; however including all three papers separately does not change the conclusion of this paper. There were two analyses that assessed impacts of refractive error on learning of students in Gansu province in 2004.^11^^,^^48^ Out of these two studies, we adopted impacts from the more rigorous study design, an RCT.^11^ Lastly, we did not proceed to benefit-cost analysis for one outcome,^57^ due to the large, reported impact on learning which was 9.5 times larger than the median impact in reported education RCTs.^59^

**Fig. 2:**
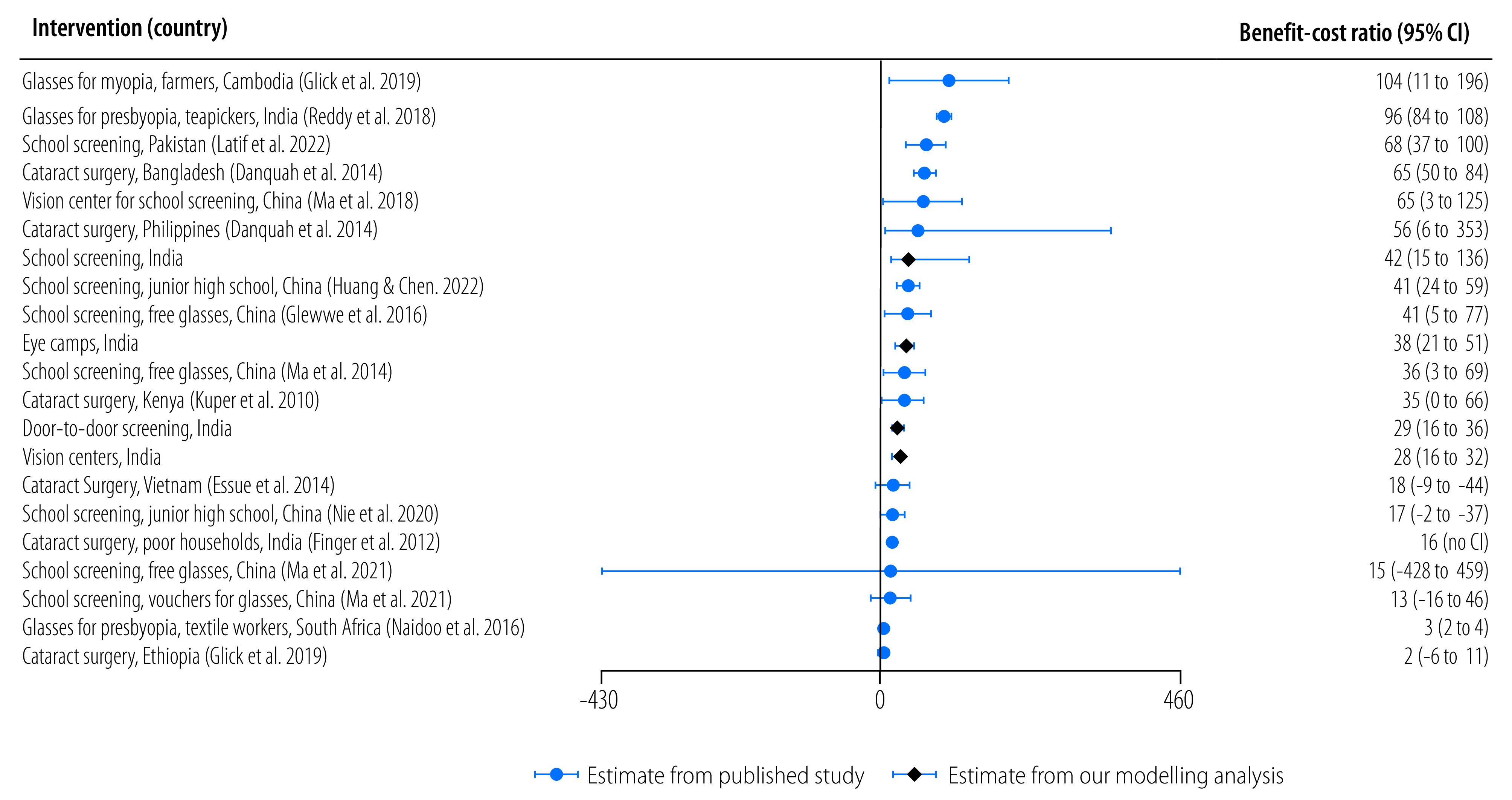
Benefit-cost ratios of eye health investment in low- and middle-income countries

The modelling analysis generated four benefit-cost ratios, for a total of 21 benefit-cost ratios. The ratios from both estimation approaches are presented in Fig. 2. Central estimates of benefit-cost ratios range from 2 for cataract surgery in Ethiopia to 104 for providing eyeglasses for farmers in Cambodia. Median benefit-cost ratio among the 21 estimates is 36 (mean: 40). The median benefit-cost ratio of 10 estimates derived from outcomes assessed as low, moderate risk of bias, or some concerns is also 36. CIs for interventions are generally large, with some studies generating CIs that contain benefit-cost ratios less than one. For the economic modelling approach, two benefit-cost ratios are above the median (school screening had a benefit-cost ratio of 42; and eye camps had a benefit-cost ratio of 38); and two benefit-cost ratios are below the median (door-to-door screening had a benefit-cost ratio of 29; vision centres had a benefit-cost ratio of 28).

CI: confidence interval.

Notes: Benefit-cost ratios derived from individual studies identified via systematic review are labelled with the relevant study. The other four benefit-cost ratios are from the economic modelling approach from scaling interventions in India. CIs in economic modelling analysis are based on Monte Carlo simulations, varying key parameters simultaneously. CIs from individual studies based on reported variation in impact parameters. Results assume a 3% discount rate.

Table 3 presents costs, benefits and results from the economic modelling approach of four interventions in India. Total costs for one year of screening activities plus follow-up costs for eyeglasses replacement over a 10- or 20-year period equal US$ 31.6 million. The total annualized cost is US$ 2.1 million. All strategies have similar costs per person treated, ranging from US$ 4.2 to US$ 6.7 depending on the strategy, with an overall cost per person treated of US$ 4.5. The annualized benefits are estimated at US$ 67.4 million, and US$ 143 per person treated per year for all interventions. The benefit-cost ratios vary from 28 for vision centres to 42 for school eye screening. The pooled benefit-cost ratio is 31.

**Table 3:** : Estimated costs, benefits and benefit-cost ratios of refractive error and cataract case-finding strategies, India, 2019–2020

Variable	Vision centre	Eye camp	Door-to-door	School screening	All strategies
**No. of patients screened**	1 373 925	307 718	286 652	332 237	2 300 532
**No. of eyeglasses dispensed**	241 454	53 325	27 413	8169	330 361
**No. of cataract surgeries performed**	74 311	54 627	13 024	0	141 962
**Cost, US$**					
Total costs for case finding	3 498 789	1 171 268	852 929	239 317	5 762 303
Initial eyeglasses cost	1 152 581	254 547	130 856	38 995	1 576 979
Surgery cost	5 276 081	3 878 517	924 704	0	10 079 302
Patient costs					
Accessing interventions	1 682 719	376 879	33 574	0	2 093 171
Surgery	2 169 881	1 595 108	380 301	0	4 145 290
Follow-up costs for repurchasing eyeglasses^a^	5 816 614	1 284 596	660 378	188 628	7 950 216
Total costs over time horizon	19 596 666	8 560 915	2 982 742	466 939	31 607 262
**Time horizon, years**	20	20	20	10	20
**Annualized cost, US$**					
3% discount rate	1 317 204	575 428	200 487	54 739	2 147 858
Per person screened	0.96	1.87	0.70	0.16	0.93
Per person treated	4.17	5.33	4.96	6.70	4.55
**Annualized benefit, US$**					
3% discount rate	37 229 768	22 033 085	5 884 621	2 272 368	67 419 841
Per person treated	118	204	146	278	143
**Benefit-cost ratio**	**28.3**	**38.3**	**29.4**	**41.5**	**31.4**

US$: United States dollars.

^a^ Net present value at 3% discount rate.

Notes: Results and intermediate values of an economic modelling study to estimate benefit-cost ratios from four case-finding interventions in India, conducted by six eye health providers during 2019–2020. Parameters are drawn from a study^32^ estimating welfare losses associated with moderate or severe visual impairment and blindness, and a microcosting analysis in India.^31^ Results based on a 3% discount rate. Values are in 2020 US$.

### Comparison to other interventions

By comparing our median benefit-cost ratio for correcting refractive error and cataract to 652 global development investments in low-and middle-income countries, we found that eye health investment returns six times more benefits (benefit-cost ratios: 36 vs 6) than the typical investment. Furthermore, our estimated benefit-cost ratio of 36 aligns with the range of benefit-cost ratios for the 12 best-buy global development interventions identified by the Copenhagen Consensus Center.^24^ The ratios for these interventions range from 18 for nutrition counselling, to 125 for electronic procurement systems. 

Compared to noncommunicable disease and nutrition interventions, our studied eye health interventions had a higher median benefit cost ratio (36) than noncommunicable disease interventions (9) and nutrition interventions (13). However, the benefit-cost ratios for single interventions showed considerable variation. For noncommunicable diseases, the benefit-cost ratio ranges from less than 1 to 102, for nutrition the range is 2 to 81, and for our examined eye health interventions it is 2 to 104. 

## Discussion

Our findings highlight that investing in eye health can yield substantial returns given that vision is integral to various societal activities, such as work, education and daily life. Improving vision has plausibly large impacts on productivity, learning and household income, while the costs of correction are comparatively small, typically around US$ 10 per year of improved vision (for non-school screening interventions) or per year of benefit from improved vision (for school screening interventions).^21^

To contextualize our results, we compared our estimated benefit-cost ratio with those of other global health development interventions. This comparison revealed that the returns from eye health investments are on par with best-buy interventions. However, the considerable variation in benefit-cost ratios for single interventions suggests that making blanket investment in domains like noncommunicable diseases, nutrition or eye health is not advisable. Instead, a careful selection of interventions and contexts that promise the greatest returns on investment is essential.

Our results are relevant for decision-makers who may be motivated by a broader goal of improving citizens’ welfare, rather than averting DALYs at lowest cost. Planning, finance and other sectors, or donors which fund multiple cause areas, should consider the potential of eye health interventions. Similarly, businesses and ministries responsible for productive sectors such as agriculture, manufacturing and services, could see vision screening as a means to enhance productivity. Additionally, the education sector might leverage vision screening to boost learning. Lastly, while eye health interventions do not typically avert DALYs at lowest cost, health ministries might consider the results presented here to justify expanded investment under broader decision criteria, as was the case for the nutrition sector in the 2010s (online repository).^21^ Furthermore, investment in eye health can reduce inequities, because uncorrected visual impairment is a determinant of poverty,^3^^,^^34^ and the benefits of eye health interventions would most likely accrue in lower socioeconomic groups.

While the results demonstrate the potential of eye health as a highly beneficial use of resources, the study has limitations. First, the evidence stems primarily from selected country and sector contexts, excluding areas like Latin America and north Africa, and some countries with high visual impairment prevalence like Indonesia. Second, many studies presented a serious risk of bias. However, the median benefit-cost ratio remained consistent at 36, even when only considering studies with a low risk of bias, a moderate risk of bias, or presenting some concerns of bias. Uncertainty surrounding the benefits is evident from the broad confidence intervals. Third, in the studies we identified, associated costs were infrequently reported. When mentioned, the focus was typically on the costs of eyeglasses or surgery, with less attention given to other substantial costs like case-finding and patient expenditures. Furthermore, some of the benefit-cost ratios in this paper are based on a recent publication discussing the costs of eye health programmes in India.^31^ Considering India’s pioneering role in delivering low-cost, high-quality eye care, it is uncertain how these costs translate to different contexts. Further research is needed to understand the variation in eye-care costs across diverse settings, ideally by examining large-scale programme implementations. 

In our economic model, the parameter of 30.2% for avoided loss of employment is from a review of 15 countries, with nearly all being high-income countries.^8^ However, the parameter is within range of a worldwide survey of 256 286 people, who noted employment losses of 21% and 36% for self-reported severe and extreme visual difficulty, respectively.^60^ Hence, the limited evidence suggests 30% loss in employment as used here is reasonable.

We assumed avoided caregiver costs to be 5% and 10% of productive time, respectively, for those having moderate and severe visual impairment and blindness. Despite the limited evidence for these parameters, we note that in a large-scale study in India, the average hours spent on caregiving for visual impairment was estimated at 4.6 hours per day, around 80% of a 40-hour working week.^61^ Studies from high-income countries indicate similar or greater hours of care required for those having blindness.^62^

This study underlines the need for further research. While our findings indicate the high potential of eye health investments, additional evidence is needed on how visual impairment affects sectors such as agriculture, manufacturing, and domestic work across different low-and middle-income country contexts. More research should focus on understanding the broader impact of vision correction, using RCTs or quasi-experimental methods. Cost estimations are equally vital, as they were often missing or incomplete in many studies. Lastly, further research should be conducted on the learning gains from correcting refractive error in school children in other countries than China.

When experimental approaches are not feasible, cross-sectional surveys comparing those with varying degrees of visual impairment with matched controls could enhance our understanding of productivity losses and caregiver responsibilities. Assessing dose–response relationships of visual acuity to productivity loss could provide more confidence in the impact of these relationships. When budget constraints hinder surveys and experimental research, identifying natural experiments and taking advantage of existing surveys will be important for enriching the evidence base.

In conclusion, this study highlights both the promise and existing uncertainties regarding the return on investment in eye health. While our assumptions are plausible, they are not precise estimates. Nonetheless, the estimated benefit-cost ratios for investing in eye health seem promising, and are comparable with other top investments in global health and development. Future research should be directed towards clarifying the areas of uncertainty. 
