# Corrigendum

**DOI:** 10.2471/BLT.24.100424

**Published:** 2024-04-01

In: Wong B, Singh K, Everett B, O’Brien KS, Ravilla T, Khanna RC et al. The case for investment in eye health: systematic review and economic modelling analysis. Bull World Health Organ. 2023 Dec 1;101(12):786–99, 

on page 794, the first entry in Figure 2 should read “Glasses for myopia, Cambodia (Sagemüller et al. 2022)”

